# How the logics of the market, bureaucracy, professionalism and care are reconciled in practice: an empirical ethics approach

**DOI:** 10.1186/s12913-020-05870-7

**Published:** 2020-11-10

**Authors:** Florien M. Kruse, Wieke M. R. Ligtenberg, Anke J. M. Oerlemans, Stef Groenewoud, Patrick P. T. Jeurissen

**Affiliations:** 1 Radboud University Medical Center, Radboud Institute for Health Sciences, IQ healthcare, Nijmegen, The Netherlands; 2 Ministry of Health, Welfare and Sport, The Hague, The Netherlands

**Keywords:** Moral limits of markets, Long-term care, Commodification, Empirical ethics

## Abstract

**Background:**

In the Netherlands, the for-profit sector has gained a substantial share of nursing home care within just a few years. The ethical question that arises from the growth of for-profit care is whether the market logic can be reconciled with the provision of healthcare. This question relates to the debate on the Moral Limits of Markets (MLM) and commodification of care.

**Methods:**

The contribution of this study is twofold. Firstly, we construct a theoretical framework from existing literature; this theoretical framework differentiates four logics: the market, bureaucracy, professionalism, and care. Secondly, we follow an empirical ethics approach; we used three for-profit nursing homes as case studies and conducted qualitative interviews with various stakeholders.

**Results:**

Four main insights emerge from our empirical study. Firstly, there are many aspects of the care relationship (e.g. care environment, personal relationships, management) and every aspect of the relationship should be considered because the four logics are reconciled differently for each aspect. The environment and conditions of for-profit nursing homes are especially commodified. Secondly, for-profit nursing homes pursue a different professional logic from the traditional, non-profit sector – one which is inspired by the logic of care and which contrasts with bureaucratic logic. However, insofar as professionals in for-profit homes are primarily responsive to residents’ wishes, the market logic also prevails. Thirdly, a multilevel approach is necessary to study the MLM in the care sector since the degree of commodification differs by level. Lastly, it is difficult for the market to engineer social cohesion among the residents of nursing homes.

**Conclusions:**

The for-profit nursing home sector does embrace the logic of the market but reconciles it with other logics (i.e. logic of care and logic of professionalism). Importantly, for-profit nursing homes have created an environment in which care professionals can provide person-oriented care, thereby reconciling the logic of the market with the logic of care.

**Supplementary Information:**

The online version contains supplementary material available at 10.1186/s12913-020-05870-7.

## Introduction

The for-profit nursing home sector in the Netherlands grew substantially in a short period of time: 50% of for-profit homes opened within the last 3 years (2019) [1]. This is not an isolated Dutch phenomenon; for-profit providers’ share of long-term care (LTC) provision grew substantially in other Western countries as well, including in the United Kingdom, the United States and the Nordic countries [2–6]. The increasing prevalence of business-oriented providers in various healthcare systems has sparked the interest of many moral philosophers [7–9]. They question whether the influence of market rationalities within the healthcare sector is desirable since commercial interests can potentially conflict with other rationalities (e.g. fairness). This reflects the wider Moral Limits of Markets (MLM) debate, which asks whether market mechanisms are an appropriate means of distributing every type of good or service. The concern is that the market for some goods may lead to an unjust distribution of goods or that the market will erode the value of the good [7, 9, 10].

This study adds to the MLM debate in two ways. Firstly, the MLM debate often discusses the contested sphere (e.g. the healthcare sector) as a whole but does not so much assess and differentiate the dynamics within that sphere. Hence, it tends to neglect the complexity and variety inherent in healthcare systems. Secondly, the MLM debate is primarily held on a theoretical level. Less is known about how values manifest themselves – and how the various healthcare stakeholders respond to market forces – in practice. This study empirically assesses these issues within the context of the MLM debate using the for-profit nursing home sector as our case study.

This study is limited to for-profit nursing homes. The nursing home sector is an especially instructive case for the MLM debate because this sector exaggerates concerns about vulnerability, solidarity, dependency and mortality. In addition, the Dutch context is of particular interest because the LTC reform in 2015 introduced market forces and boosted the creation of a for-profit market for individuals to choose and organise their own care. We look specifically at the for-profit sector because we postulate that for-profit nursing homes are particularly influenced by the market logic compared to the public and non-profit homes. Hence, we argue that this sector offers a valuable case for studying how the four logics are balanced, prioritised and reconciled in practice. Given the recent growth of for-profit provision nursing home care in the Netherlands, it is of particular policy interest to put these for-profit homes under the moral microscope. In a similar vein, the increasing commercialisation of the LTC sector in the Netherlands could offer new insights for the MLM debate.

Some important features of the Dutch for-profit nursing homes are worth pointing out in advance. The nursing home sector has historically been dominated by non-profit nursing homes. The Dutch LTC sector does not have public nursing home providers. For-profit nursing homes are relatively small: the average number of clients in the traditional non-profit sector is 64, while there are 19 clients on average in for-profit nursing homes [1]. There is a lack of quantitative information about case-mix differences between for-profit and non-profit nursing homes, but there are indications that for-profit nursing homes have a lighter case-mix compared to non-profit nursing homes [11]. Furthermore, for-profit nursing homes tend to serve a more affluent clientele [1, 11]. Additional File A provides a detailed description of the institutional background of the LTC sector and how the number of for-profit nursing homes in the Netherlands has grown.

In order to have a theoretical tool to analyse our empirical findings, we constructed a theoretical framework by compiling and synthesising previous theoretical contributions on this topic. The theoretical framework defines different ‘logics’ and their respective values to refine and sharpen the MLM debate. Logics are defined as laws of thought or rationales behind practices. (The term ‘logics’ was also used by Annemarie Mol [12, 13]). These logics are important for the MLM debate because it enables discussion about the limits of the market sphere [10]. In our study we define four logics: the market, bureaucracy, professional and care logics.

The central questions of this study are: 1) how are the four different logics reconciled in practice?; and, 2) which logic is dominant for each of the stakeholders (i.e. experts on the for-profit nursing home market, nursing home managers, care workers, family members of residents, and residents)? Special focus will be placed on how the logic of the market influences practices within the for-profit nursing home sector and how this is combined with the three other logics (bureaucracy, professionalism and care).

## Theoretical framework

### Four different logics

The theoretical framework consists of four logics. Three of the four logics are based on the contribution of Freidson [14]. He contrasted the logics of the market, bureaucracy and professionalism [14]. We consider the logic of care, defined by Mol [12], to be a necessary addition to the framework to capture the relational process of caring.

Although Freidson’s analysis is sociological and Mol’s analysis is ethical, we argue that they can be brought together in one theoretical framework. Freidson’s logics – the market, bureaucracy and professionalism – are not morally free, they carry moral codes, values and motives. (Sociology and ethics are closely tied for good reason [15].) Moreover, we follow an empirical ethics approach (more on this later) which means that by integrating empirical social analysis with ethical analysis, we can draw normative conclusions [16]. Hence, the two disciplines are already integrated by means of this approach.

In order to carefully unite the different contributions, we distil four dimensions of each of these four logics which help us uncover and categorise the ethical foundations of the care relationship. The first dimension, ‘values’, considers which values are central to each logic. The second dimension, ‘care as’, relates to how each logic views the activity of care provision. The third dimension, the ‘care relationship’, concerns the relationship between care provider and care recipient. And the fourth dimension, ‘motive’, addresses the motivation of the care provider according to each logic. These theoretical classifications are simplified and outlined in an overview in Table 1.

**Table 1:** The four logics

Logic	Market	Bureaucracy	Professional	Care
**Value**	(Negative) freedom, autonomy, rationality, choice	Accessibility, rationality, control, thoroughness	Trust, collective knowledge, quality	Relationship forming and stabilising values (e.g. generosity, forgiveness)
**Relationship**	Commercial: impersonal, equal, fungible, demand driven	Impersonal, hierarchical, rational	Hierarchical	Interdependent, personal, equal (in their moral rights)
**Care as..**	Commodity	Procedure	Higher professional goal/Discipline	Process
**Motive healthcare provider**	Profit maximising	Equal treatment	Intrinsic	Emotional/Social

Based upon the following sources: [8, 13, 14, 17–23]

### Framework of the four logics

Table 1 serves as a framework with which to understand the different logics in practice. This table unites and builds upon insights from existing theoretical literature.

The process of assembling the different perspectives on the MLM was done by means of an extensive literature study and by having regular group discussions to select the concepts and dimensions for our framework. The benefit of this theoretical framework is twofold. Firstly, it provides a clear and comprehensive overview of the different positions that have been juxtaposed in the MLM debate. Secondly, it serves as the analytical lens through which to view our empirical findings; and, to be more specific, it helps us to consider the ways in which these different logics are balanced with one another.

The remainder of this section provides a description and overview of each logic; and with each logic, it provides a description of the different dimensions.

### Logic of the market

Adam Smith, a philosopher by training, lay the foundations of classical economics by describing the benefits of the division of labour and the free market [21]. Smith theorised that ‘the invisible hand’, the pursuit of self-interest of people within a competitive market, is beneficial to the public interest. Around the 1980s, this ideology spread to the healthcare sector in parts of Europe and Asia with the objective of enhancing efficiency and improving quality of care [24–26].

#### Values

The core values of the free market are rationality, efficiency, responsiveness to need, and innovation – all to increase profit. Moreover, the buyer and seller should fully enjoy their freedom to choose – only bounded by the limits of the law – because the idea is that only the person in question can know what they want or need. Within the market logic, this belief in the power of ‘choice’ is paramount [12] and requires (negative) freedom [17]. (Negative freedom refers to the absence of obstacles or interference from others to be left to do or be able to do what they desire to do [17].) Moral deliberation prior to the act of choosing is a private concern [13].

#### Care as

The market logic treats care as a product that can be traded on the market. In other words, the provision of care is commodified [8, 19]. This implies that the value of care is fully expressed in monetary terms; it does not possess a social meaning. Consequently, the product is fungible. In addition, the logic of the market considers care-provision as property.

#### Relationship

The logic of the market is based on the idea that buyers and sellers act as *homo economicus*: someone who makes rational decisions out of self-interest [27]. According to this reasoning, individuals are driven by the maximisation of their own utility; in other words, the logic of the market is associated with individualism and consumerism. (Foucault (1978) argued that the order of causality is reversed: the market shapes individuals to be self-interested and rational human beings; they become dependent on the logic of the market because the market logic has shaped them to think this way [18].) The relationship between the buyer and the seller is a commercial one [19]. It is impersonal, fungible, instrumental, rational and relies on an equal relationship. The relationship is demand-driven, which means that it is responsive to the wishes and needs of consumers. The market logic is based upon the notion that all the stakeholders involved in the care transaction make economic and political choices that will serve their best interests. Hence, consumers are always right in making their own decisions and they do not need specialists to choose on their behalf [14]. The clients are selected by their ability to pay and through contractual agreements the relationship is sealed.

#### Motive

The driver behind the logic of the market is that organisations strive continuously towards profit maximisation to satisfy their shareholders. In order to maximise profits, organisations have to raise revenue and optimise efficiency. To achieve the former, the main objective of the organisation is to satisfy their clients in order to keep existing clients and possibly attract potential clients. To optimise efficiency, the organisation will try to minimise the marginal costs and optimise the use of fixed-cost resources. The organisation constantly seeks to achieve the equilibrium between optimising efficiency and maintaining the quality of their product (as any loss of quality might deter clients from purchasing their product).

### Logic of bureaucracy

The logic of bureaucracy originates from the intellectual legacy of Max Weber [20]. The bureaucratic rationale was developed after the industrial revolution when large-scale and complex organisations emerged. Organisations in the industrial period had to be run by different principles than traditional decision-making tools, which was driven by traditional authority – i.e. making decisions based upon kinship, relationship and particularism. However, new large-scale organisations demanded rationalisation, formality, specialisation and hierarchy [28].

#### Values

The core objective of bureaucratic logic is to treat clients equally. In addition, organisational control is central to bureaucratic logic as the means to minimisation of risk and maximisation of accountability. Hence, bureaucratic organisations endorse values such as rationality, carefulness, thoroughness, lawfulness and predictability.

#### Care as

The bureaucratic logic considers care to be the highly organised and systematised provision of care to citizens in a non-discriminatory fashion, with all receiving the same care and all treated equally – and with little or no room for customised care [14]. Furthermore, the logic of bureaucracy understands care as a linear system of multiple care processes such as washing and feeding. In other words, care is not a single act but a system involving many procedures. The central objective of the logic of bureaucracy is to organise the process of care; outcomes are secondary.

#### Relationship

The logic of bureaucracy defines the care relationship in terms of rationality, predefined procedures and laws because the bureaucratic organisation has to be impersonal to protect itself from particularism [28]. Hence, the relationship is impersonal and hierarchical. The care seeker counts on universal access, availability and quality of health care services. However, the care seeker is only entitled to services as prescribed and cannot influence his own care or be an active co-producer of care.

#### Motive

The motivation that drives bureaucratic organisations is that they want to maintain or improve their rational functioning. Any loopholes or flaws in the functioning of the bureaucratic organisation will be addressed by modifying existing procedures and laws or by implementing additional formal rules.

### Logic of professionalism

Freidson (2001) defined the logic of professionalism as follows, “In the most elementary sense, professionalism is a set of institutions which permit the members of an occupation to make a living while controlling their own work” ([14], p.17). Professionalism rests on two beliefs: (1) the belief that with only the required training and experience, professionals can perform their specialised work; (2) the work of professionals cannot be standardised, rationalised or commodified [14]. Only the professional has the specific, tacit, almost esoteric knowledge to do their work. For care provision to be effective and optimal, the professional needs a professional space that establishes favourable economic and social conditions, allowing the professionals to control their own work [14].

#### Values

The professional relies on trust from all stakeholders involved (i.e. managers, inspectors, recipients of the service of the professional) because only with trust can professionals execute their work. In addition, one of the central values of the professional logic concerns the acquisition of knowledge and sharing of knowledge among peers [29].

#### Care as

The professionalisation of care is creating a discipline: ‘care-as-discipline’ [30]. This means that care is systemised through the need and control of formulating theory and the fabrication of knowledge concerning ‘care’ [31]. The professional care logic expresses ‘good’ care provision by complying to norms and standards defined within their professional field [32]. Therefore, in theory, the provision of care is at its best when the professional is given its full autonomy.

#### Relationship

The relationship between professional and client depends on who has permission and legitimacy to exercise control [32]. The theory is that the care recipient is dependent on the professional; they are the beneficiary of professional knowledge and of the skills of the care provider. Therefore, the relationship becomes hierarchical. Professionals are less bound by rules and enjoy more freedom to make decisions based on their professional title compared to working in a bureaucratic organisation. In other words, the relationship is built on trust – trust that the professional has the best motives and intentions to provide the optimal care.

#### Motive

The professional is the main driver of the organisation. Importantly, the professional is intrinsically motivated to provide the best care. The purpose of their profession is to serve the patient’s needs (not simply what the patient wants or what they can pay for, in contrast with the market logic) [29]. Furthermore, in theory, the professional’s work is about more than making a salary: work becomes their life and their identity. According to the logic of professional assumes that satisfaction is largely gained from perfecting their performance. “[S]atisfaction is intrinsic to the performance of work that is interesting and challenging because it is complex and requires the exercise of discretion” ([14], p.108).

### Logic of care

Although various classical philosophers analysed the concept of care (e.g. Aristotle, Descartes, Kant) [33], Carol Gilligan was the first, in 1982, to coin the term ‘ethics of care’ [22]. Gilligan rejected the Enlightenment notion of humans and human relationships as purely rational, as embodied by Kantian universalist ethics, and argued instead for a ‘care perspective’ which acknowledges the role of emotions. The ethics of care instead emphasises the importance of situation-specificity, interdependence and emotional sensitivity. Relatedly, Annemarie Mol [12, 13] introduced the term ‘logic of care’ as a critique of the values imposed on the care relationship by the market.

#### Values

The logic of care treats caring itself as a virtue [34]. Because relationships are central to the logic of care, values such as sharing, mutual respect, responsibility for one another, and genuineness are particularly important. However, different values are important in different contexts so no single, rigid set of values can be formulated for all situations.

#### Care as

The logic of care is distinguished in several ways from the other three logics. (i) The logic of care rejects the notion that we can rely exclusively on rationality to solve moral problems. Care ethics is instead informed by emotional wisdom – intuition, inclinations and feelings. (ii) The logic of care is built on the notion that people are fundamentally dependent on other human beings. Interpersonal situations necessarily involve dependency relationships. (iii) The logic of care acknowledges that care is a process involving the care recipient and the caregiver; it is not a compartmentalised procedure (as in the bureaucratic logic) or linear transaction (as in the market logic). In the logic of care, care is not a means to an end, nor is it instrumental (as in the market logic). Instead, the process of caring is an end in itself [13]. (iv) The logic of care is situation-dependent and therefore the appropriate care is determined on a case-by-case basis and not by bureaucratic or (moral) universalist rules.

#### Relationship

For other logics, ‘care as’ and ‘relationship’ are distinct dimensions. By contrast, the logic of care is centred entirely on the ‘care relationship’. Whereas in other logics individuals are treated as independent and rational beings, in the logic of care, individuals are treated as interdependent and shaped by their relations with others. Different from the logic of bureaucracy and professionalism, the logic of care distances itself from a hierarchical relationship between the professional and the care recipient and advances a more equal relationship.

#### Motive

The motivation that underpins the care relationship can be defined as a gift good [8]. Gift goods cannot be expressed in purely monetary terms. Instead, the value of the gift goods derives from factors other than market value, such as friendship and respect [8]. The ‘rewards’ of caring are described as transformative because in the care relationship both caregiver and care receiver are cultivated [35].

### Bridging the gap between theory and practice

The four logics are ideal types, used for instrumental purposes to analytically assess the phenomenon in practice. (See Table 1 for a brief overview of the four logics.) In order to bridge the gap between theory and practice, we first need to acknowledge that different logics co-exist in practice – similar to the idea of ‘complex pluralism’ [36]. However, the interplay between the different logics is of interest; one logic can dominate over the others. This can depend on, among other things, the conditions wherein stakeholders are incentivised to pursue a certain logic. For instance, a nursing home manager can uphold primarily professional values but in financial distress the market logic might overrule.

Many scholars have written about whether market forces undermine and supress other valuable logics in healthcare [7, 9, 19]. Two main objections against market forces in healthcare are that markets perpetuate inequalities and markets degrade the value of a certain good – or in other words, it can corrupt the good [7, 9, 37].

In that vein, the desirability and impact of commodification of care has received much attention in the MLM debate. Pellegrino (1999) outlines why the market ethos might not be suitable for healthcare services, and should not be commodified, by comparing the characteristics of care to the definition of market goods. Pellegrino argues: (i) healthcare provision is not fungible; (ii) providing healthcare services is not a possession; (iii) the provision of healthcare is a personal relationship; (iv) the nature of illness and the healing process are not products which patients can consume and which the doctor produces out of materials [19].

Kaveny [38] and Radin [39] contribute to the commodification debate. Kaveny [38] argues healthcare has three different purposes (i.e. public health purpose, measurable health improvements and the non-measurable individual health improvements) and, hence, is a polyvalent good. The extent to which, and how, commodification affects each purpose differs [38]. Similarly, Radin [39] argues that a ‘spectrum of commodification’ offers a solution for the management of market forces in healthcare. According to Radin [39], market and care provision could overlap and co-exist without seriously eroding each other. This theory of ‘incomplete commodification’ could lead to different solutions to reconcile the market and the provision of care. For instance, to shield the healthcare sector from being entirely commodified, the healthcare system should not merely rest on the market-based philosophy of incentives, and should avoid the exclusive use of market-based terminology [38].

The impact of the logic of the market on the logic of professionalism has been a matter of interest too. Freidson [14] argued that professionals can uphold their values in spite of market pressures if they are able to maintain the dominance of their profession in the provision of their services (e.g. ensuring professional certification as a condition of employment) and can force social closure (i.e. build exclusive communities in order to monopolise scarce resources for their own professional group). However, others have pointed to ways in which both market and bureaucratic forces influence and change professional practices [40]. Authors including Light [41] and Reinhardt [42] are more sceptical about the altruistic or civic values of healthcare professionals and focus instead on healthcare professionals’ attraction to markets and corporations that advance their interests. “They [physicians] are as decent as other human beings, and just as frail under severe economic pressure.” ([42], p.22).

The logics of the market and bureaucracy seem to be more intertwined and less contested than the interaction between the other logics. The logic of the market and the logic of bureaucracy co-exist in almost all markets in practice. Bureaucratic mechanisms have long been used to tame market failures. Yet, the degree of bureaucratic penetration strongly varies. The risks of market failures are higher for public goods or social services of general interest and, hence, the market needs more fine-tuning through regulation [43]. Nobel prize winner Kenneth Arrow [44] argued that the healthcare market will never be able to function according to pure market logic. Likewise, Adam Smith was aware of the possible market failures and argued for strong supportive social institutions [21, 45]. The market of healthcare has several characteristics that distort the mechanisms of the market. Firstly, for example, the nature of demand is irregular, unpredictable and “with an assault on personal integrity” ([44], p.949). Secondly, there is uncertainty about the quality of the product that patients purchase on the healthcare market. Because of these inherent market imperfections in the healthcare system, the Netherlands has adopted a regulated healthcare market [46], which is to say the Netherlands implemented a hybrid form combining market principles and bureaucracy. Nonetheless, there is still friction between the logic of the market and the logic of bureaucracy because bureaucracy is rigid and depends on regulation whereas flexibility and deregulation are two of the vital conditions for market mechanisms to function efficiently.

However, all the aforementioned theoretical nuances still take the healthcare sector as a whole; they do not differentiate between the different healthcare system levels. This study, therefore, takes a multilevel approach, in order to add some depth to the theoretical framework that underlies the MLM debate.

## Method

### Approach

This research takes an empirical ethics approach to the topic [47, 48], following the critical applied ethics method [49]. The methodology is phenomenological, involving both deductive and inductive work. Phenomenological ethics is about studying “moral perception and reflective subjectivity of real, situated persons” ([50], p.443), and this study seeks to do exactly this. Our primary source of information is qualitative data derived from interviews; our observations made during the course of fieldwork were used only to connect the dots between the qualitative findings.

This study takes a multilevel approach, distinguishing between the levels of: (i) the individual care relationship (micro), (ii) the care organisation (meso), (iii) and the healthcare system (macro). The micro-level relates to the values that shape the relationship between the care recipient and the care provider. The meso-level concerns organisational and institutional values at the level of the healthcare organisation. The macro-level refers to societal values and includes characteristics of the healthcare system in general. It is important to take a multilevel approach because values exist at different levels (i.e. micro, meso and macro-level) and the values are often interconnected across levels [51]. Failing to study these values on different levels might lead to potential problems of misspecification, aggregation bias and contextual fallacies [51].

### Data collection

#### Interviews

We conducted semi-structured interviews guided by the following questions: (i) How would the respondent define ‘good care’? (ii) Does their respective nursing home achieve their notion of ‘good care’; and, if they do achieve this, how does the nursing home do so; or, if they do not achieve this, why don’t they? (iii) Which activities are associated with achieving ‘good care’? We asked all participants explicitly to provide examples to illustrate their answers. The topic list also included stakeholder-specific questions (i.e. residents and family members of residents, employees, managers, experts). (The topic lists, including stakeholder specific questions, are outlined in Additional File B.) The interview guide for the experts concentrated on the role of for-profit nursing homes in LTC provision.

#### Sampling of cases

This study selected three for-profit nursing homes located in different provinces and regions (rural and urban) as case studies. We would like to stress that it is beyond the scope of this study to draw a comparison between the for-profit sector and the non-profit sector. However, we do report on how the for-profit sector perceives the non-profit sector. This narrative is important for understanding how for-profit homes define their role as LTC providers.

The participating nursing homes were selected by means of purposeful sampling, based on their organisational characteristics, in order to cover a wide spectrum of the for-profit market. One home is financed through personal budgets; one through total home-care packages; and one was formerly a personal budget financed home but became financed by means of total home-care packages. (As mentioned previously, for more information about the financial reimbursement schemes, please refer to Additional File A.) Another difference is that two are part of franchises and one nursing home is a stand-alone home. We hypothesise that the balancing act of the different logics could differ between the different types of nursing homes because personal budget homes rely entirely on private transactions whereas nursing homes financed with total home-care packages only partly rely on private contracts.

#### Sampling of respondents

We purposively selected respondents from the different system levels. Individuals from three groups were selected in each caring home: 1) residents and/or family members, 2) employees and 3) nursing home managers (see Table 2). For every nursing home we included at least two residents and/or family members, two employees, and at least one manager. The nursing home managers could also be the owner of the nursing home. (We do not distinguish between nursing home owners and nursing home managers in this article; both are referred to as nursing home managers.) In addition, we selected a wide range of experts (e.g. branch representatives, consultants, other home managers, government officials) in order to collect multiple perspectives on the macro-level.

**Table 2:** List of respondents

		N	Level
**Total**		35	
**Experts (including other nursing home managers)**		15	Macro
	Director/staff for-profit nursing home (chain-affiliated)	3	
	Director/staff for-profit nursing home (stand-alone)	3	
	General sector expert	5	
	Institutional actor	3	
	Director/staff non-profit facility	1	
**Location managers of one of the three for-profit nursing homes included in the case study**		4	Macro/Meso
	Owners	3	
	Manager	1	
**Employees**		7	Meso/Micro
	Nurse	5	
	Other employee type	2	
**Residents or family members on behalf of the residents**		9	Meso/Micro
	Residents	6	
	Family of residents	3	

After approval and support from the manager of the nursing homes, the researchers recruited respondents when the researchers were on site. (On a few occasions the employees or managers assisted us to the residents with only a mild or no cognitive impairment to ask them whether they wanted to partake in our study because it is difficult to assess as an external researcher who is cognitively capable to be interviewed.) We only interviewed respondents who were capable of informed consent. On a few occasions, we assessed that the respondents were not able to do so after a brief informal conversation. In two of the three homes, a newsletter was sent out to the residents and their family members to inform them about our visit. This letter invited family members to share their views with us.

We did not distinguish between the different types of employees. We included a variety of employees, ranging from senior nurses to activity organisers. We define all these employees as professionals in their own right. Hence, the term ‘professionals’ includes a full range of professions working in for-profit nursing homes.

All respondents gave their informed consent. We conducted 35 interviews and the duration of the interviews ranged from approximately 20 min to over an hour with an average of 33 min. As confirmed by the medical ethical committee, (file number 2019-5256) this study does not fall under the scope of the Dutch Medical Research Involving Human Subjects Act (WMO), as our study did not involve subjecting participants to procedures or rules of behaviour that may infringe the physical and/or psychological integrity of the study subjects. This study instead follows the Netherlands Code of Conduct for Research Integrity which is similar to the European Code of Conduct for Research Integrity [52].

### Data analysis

The interviews were recorded, transcribed verbatim and afterwards analysed using qualitative analysis software program ATLAS.ti. The a priori codes, deducted from the theoretical framework, were used to categorise the data; the newly-identified codes emerged from the data itself. Two researchers independently coded the interviews. All codes were checked by the other researcher. Inter-coder reliability was improved by numerous discussions throughout the coding process between the two researchers. Often the respondents were explicit in their use of terminology and fitted one of the four logics accordingly. Disputable records were discussed among the researchers. In order to select the key themes from our evidence, we used as a guide the number of times the themes were mentioned and how many respondents mentioned those themes. The key themes were determined based upon consultations between the various researchers, and by using tools such as creating a visual representation of the codes and their respective connections.

#### Reporting

When reporting the findings, to improve the readability of this article, we only mention specific stakeholders when there is no general consensus among the different types of stakeholders on the subjects we discuss in our findings. Furthermore, the results section refers to narratives instead of logics because the results section outlines the narratives of the interviewees. The discussion then reflects on the relationship between those narratives and the theoretical framework (i.e. the four logics).

## Results

### Categorisation

We inductively categorised our findings into four main themes: 1) the for-profit nursing home environment; 2) the professional in the for-profit nursing home; 3) the residents; and 4) system levels.

### For-profit nursing home environment

People described for-profit nursing homes in two ways: some respondents described the for-profit nursing homes as a place that feels like ‘home’ (mainly mentioned by employees and the managers), other respondents (solely experts) described for-profit homes as ‘hotels’. Residents generally refrained from depicting the entire nursing home in a certain way. A few residents restricted their description to their room, which they described as their own personal space.

Most respondents described the traditional (non-profit) nursing home as the antithesis of the for-profit nursing home: the traditional nursing home was depicted as ‘the bureaucratic medical institute’. Often, respondents drew comparisons with the non-profit sector to describe their own position although none of the questions (except the questions for the experts) were designed to elicit such comparison.

The respondents described that, for them, for-profit nursing homes provide a different (and better) environment to the non-profit sector. The for-profit nursing homes are small-scale homes, with a maximum of 25 people, compared to large-scale traditional (non-profit) nursing homes. Often, the for-profit nursing homes are located in nice (historical) buildings. We found that the environment matters in five different ways.

Firstly, one of the most important conditions that the small scale of for-profit nursing homes provides is *time*: time to provide care. This condition was mentioned very often and emphasised during the interviews. Sufficient time for healthcare professionals to provide their care was perceived as one of the key factors for good quality of care.

*Well, they can just say how they want it. And I believe it’s very important to listen to this. You also have those larger houses where they usually wash all people in the same way. But we really try to listen to the person to see what exactly they want, you know? [..] They can just indicate it. You just have the time for it. I very much appreciate that.*

Employee.

Secondly, because these nursing homes are small-scale entities and there is ‘time’ for the care relationship, there is room for person-oriented care. This entails being responsive to the different wishes of the residents and taking time to listen to the stories of the residents. Person-oriented care gives residents the feeling they are acknowledged and ‘seen’.

[Response to the question ‘what is good care?’. Later the respondent confirmed that this nursing home adheres to his/her vision]

*That you have a good sense of what people mean. [..] Sometimes you have to encourage them. Sometimes it comes naturally that they talk. [..] In addition, you have to understand the condition that someone is in. What is his[her] physical condition, his[her] health, what is his[her] religion and what does he[she] not believe in. These kind of things. Good care. That you are not indifferent to him[her]. That you sense what he[she] means. And that you are eager to know what that is. It is very important that you would like to know.*

Resident.

Thirdly, for-profit nursing homes seek to create an environment in which residents can be ‘themselves’ and sustain their usual way of living with as few modifications as possible. All the different stakeholders of the for-profit nursing home emphasised that they find it important that the different daily rhythms of the residents are accommodated. For instance, if a resident wants to wake up at seven in the morning, the manager will try to ensure that an employee is there to help this person out of bed, just as much as if someone wants to wake up at ten. In addition, when possible, the employees will keep doing the ordinary daily activities with the residents (e.g. going out for grocery shopping).

*It’s just small scale: cooking together, eating together, being able to go outside whenever you want. It all sounds very normal, but it really isn't.*

Employee.

Fourthly, non-profit nursing homes were used as an antithesis, and was characterised as an institution which is too much focused on process – i.e. rules and checklists – and not on outcome. The respondents ‘accused’ the non-profit homes of using rules as means-end. Too little time was spent on providing the actual care. The respondents characterised the for-profit nursing homes as the opposite of this: more outcome oriented. The main objective of for-profit nursing homes is that the resident is happy and satisfied. (The latter was solely mentioned by managers and experts.)

Lastly, respondents (primarily experts) depict traditional (non-profit) nursing homes as being too fixated on minimising risks. The experts describe that this pursuit to control the situation is realised by bureaucratising their nursing homes. However, according to the respondents, this comes at a cost of aspects of human dignity, such as freedom of mobility and the joys of life (e.g. drinking alcoholic beverages). For-profit nursing homes embrace the idea that risks are inherent to human life and, according to the respondents, only through the acceptance of risks can a dignified way of living be achieved.

*When people come here for a tour I say this: "we have a staircase, we have an open door, those are certain risks that we take". But you can't live without risk. If you want to live without risks, then you have to start building prisons. Life without risks is really not more pleasant, that's a lot more unpleasant in fact. In the four years that we have now been open, we have had once that someone got out and we did not know until someone called, ‘Hi, this gentleman is walking here, I think he lives with you’. That happened once. And otherwise, people want to go out very often, then you walk with them for a moment, and when they [the residents] are at the end of the path they [the residents] say it is far enough. ‘Shall we now go back?’ Then all the restlessness is over.*

Nursing home manager.

### For-profit nursing home professionals

Similar to the nursing home environment, professionals in for-profit nursing homes are contrasted with professionals in non-profit nursing homes. Our qualitative data indicate six factors that define the for-profit nursing home professional.

Firstly, the respondents define the for-profit nursing home professional as less medically-oriented and process-driven, and more focused on wellbeing (primarily mentioned by the managers and experts), compared to the non-profit sector. One of the managers referred to the wellbeing approach as the ‘happiness approach’.

Our respondents (mainly the experts and nursing home managers) argued that nursing training directs its efforts at the wrong things. Nurses learn to work hard to complete all the tasks are demanded of them, ticking all the boxes, and to obtain useful (medical) knowledge, but they do not acquire the ‘tacit’ art of ensuring the wellbeing of the residents. For-profit nursing homes actively recruit professionals who are more inclined to embrace the wellbeing approach. The essential characteristics for the professionals working in for-profit nursing homes are patience, eagerness to learn (these two were mostly mentioned by managers), commitment and passion for the job. Several nursing home managers mentioned especially that the nurses with more experience (i.e. often older nurses) and people coming from other service-oriented businesses are often more suitable to fulfil their ideal of a professional in the for-profit setting.

*Well, what we also find important is that the caregiver doesn't just want to do caregiving tasks, you know? [..] Wellbeing is very important. But in their education, a little more attention is paid to that recently, but for a long time it has been neglected. [..] What you notice then is that wellbeing, to offer that to residents is quite work-intensive. There are a number of employees who find it difficult [to adopt the wellbeing approach] and who try to avoid it. Especially in the beginning, we sometimes said: "Guys, that kitchen counter has been cleaned six times now, just sit down with the residents." But then they [employees] feel that they are not working hard. You notice that the qualified employees are trained to work very hard and when we say: “Yes, but you know? Playing a board game with seven people with dementia is much harder work than getting three people dressed,” they find that very difficult to accept because they really feel that they are not working then.*

Nursing home manager.

Secondly, the respondents observe that large scale (non-profit) nursing homes promote the idea that everyone is equal and that everyone should get the same treatment, whereas for-profit organisations like to profile themselves as homes that follow a person-oriented, customised approach. The professionals in for-profit nursing homes are able to provide person-oriented care because they enjoy professional discretion to make their own judgements and act accordingly.

*Yes, we also offer specific care to people. Not all the same, but really all exactly the care that is needed for them.*

Employee.

Thirdly, according to the respondents, the professional can only provide person-oriented care when the professionals are less subject to bureaucracy and hierarchy. Managers and experts distance themselves from the idea of strict division of labour; their personnel should respond to the wishes of the residents, irrespective of what their professional title dictates. (This was solely mentioned by managers and experts.)

*Within [our private nursing home] I deploy all employees around the care for the resident. So also the cook is an integral part [of the nursing homes] or the handyman [..] What I see in the larger nursing homes, larger institutions, is that they very much think in layers. Facility services only cleans, nursing only do their nurses tasks, the cook arranges food. And I think that everyone who works in healthcare or elderly care also works for the resident. So for me it does not really matter that the cook walks to the elevator with one of the residents when the resident no longer knows [where his/her apartment is]. [..] What I find important, of course, is that the washing and dressing, medication [part], is done by someone who is trained. Let that be clear. But over the course of the day, I don't think that [an employee’s job description] is very important anymore.*

Nursing home manager.

Fourthly, the for-profit nursing home professional is seen as a professional who is passionate about their work. Various examples were given by residents or family members of residents in which the professional would assist the residents outside their normal working hours. In their spare time they might, for example, do additional work to improve the quality of life of the residents.

*An initiative from [this nursing home] has been to bring [our resident family member] back to her birthplace in Friesland. That was an initiative of two employees. They got the car from [the nursing home] and in their own free time they went to Friesland with her.*

Family member of a resident.

Fifthly, some employees mentioned that they strive towards a more equal care relationship. They share their own life stories with the respondents. They want to acknowledge that they are allowed to come into the private sphere of the residents, and by sharing their own story they want to express a certain reciprocity. However, according to the stories of the residents and the employees, the residents did not necessarily express similar interest in the lives of the caregivers.

Lastly, from the perspective of the professional and nursing home manager, the vision of the for-profit nursing home professional is that the residents have to be mentally stimulated by the professional. They argue that otherwise the cognitive functions of the residents will deteriorate.

*“What can someone still do themselves? I also think it is good care that you encourage someone to do those things [..] Yes, I believe if you know that someone can still do certain things, for instance, brushing your own teeth. Then you give someone the toothbrush and then I say "you start" and then often with that action, that realisation comes again from "oh yes". And when you do that calmly, it often works.”*

Employee.

### The residents

Most managers and experts suggested that the current residents of nursing homes belong to a new generation with different demands and attitudes compared to earlier generations. It seems that the current generation of residents embrace the narrative of the market more strongly. Values such as individualism, private responsibility, freedom of choice, and autonomy were mentioned as important values by all types of respondents. The idea that residents value and make use of the narrative of choice is supported in two ways. Firstly, they made a conscious choice in their selection of a nursing home. In fact, some respondents said that they deliberately moved from a non-profit home to a for-profit home. Secondly, the residents explained that they value the freedom to determine their own daily rhythms, activities and living arrangements (e.g. they are free to decorate their own space). For example, residents highlighted the importance that they should be free to choose whether they want to join dinner with the other residents or to stay in their rooms.

We find that the for-profit market is less successful than it would like to be in creating a community within their nursing homes. The residents expressed conflicting ideas and emotions about living in a group. Some of them highlighted the tension between individual freedom and living in a community setting.

*It is nice here, but [as an example] there will be music tonight and I would like to sit in my own place. Because then I am close to the music, I like that so much, you know. At one point, [inevitably] you have to leave your seat, and in the meantime other people take your place. There is nothing you can do about it. And then you have to look for another place to sit, you know. I think about these things, you know, that's one of the reasons why I sleep poorly.*

Resident.

Residents often mentioned the lack of belonging and the lack of meaningful relationships with other residents. One of the reasons suggested by the residents themselves, and also observed by the researchers, is the wide variety of care needs among the residents. The residents expressed in the interviews that they missed a social connection with the other residents suffering from severe memory loss (often dementia), possibly because the residents participating in this study had only mild, or no, cognitive impairment.

*Respondent: It was a bit disappointing to me, the different types of residents here [the nursing home]. The number of people with dementia is high here. And I find it difficult to make contact with [them]. That was very disappointing to me.*

*Interviewer: And did you know this beforehand?*

*Respondent: No not really. I have the feeling that it was presented a bit nicer to me then how it really is. But it depends how you look at it, no?*

Resident.

Some residents highlighted the importance of social belonging within nursing homes (e.g. religious background). Hence, the disconnection between people within nursing homes was also attributed to the fact they came from different social groups. This feeling contrasts with the desire expressed by the managers to build a ‘home’.

### System levels

Our findings indicate that the balancing of the four narratives differs among the different stakeholders.

We found that experts (i.e. macro-level) primarily adopted a market narrative. Private responsibility and freedom of choice was valued highly. In addition, according to the experts, contractual agreements are the important binder between the nursing home manager and residents. The experts explained that this should empower residents to hold the nursing homes accountable when care and other services are not delivered to the agreed standards. The experts explained the rise of for-profit nursing homes and the demand for their services as due to the for-profit sector’s responsiveness to the wishes of the clients, which illustrates the demand and supply rationale of the market narrative. Furthermore, residents were typified as ‘customers’ with individualistic demands.

The nursing home managers (i.e. meso-level) expressed mixed values. They also spoke according to the narrative of the market – ‘they have to run a business’ – but, in addition, some expressed an interest (i) in trying to build a ‘home’, and (ii) in embracing person-oriented care. The managers of all three for-profit nursing homes demonstrated personal knowledge of their residents and their specific character traits.

The employees and the family members of the residents (i.e. micro-level) embrace values that fall under the narratives of care. However, the residents and the family members of the residents expressed various market values: they valued autonomy and freedom of choice highly and showed little interest in the reciprocity of personal relationships with their caregivers.

## Discussion

The aim of this study was to answer two main research questions: 1) how are the four different logics (i.e. the logics of the market, bureaucracy, professionalism and care) reconciled in practice?; and, 2) which logic is dominant in the narratives of each of the different stakeholders (i.e. experts on the for-profit nursing home market, nursing home managers, care workers, family members of residents, and residents)?

### The for-profit nursing home environment

Our findings suggest that it is not so much the care relationship that is commodified but that the nursing home environment and the conditions provided by the nursing home are the main commodities to be purchased on the market. According to the respondents, for-profit nursing homes create an environment that enables the professional to execute their profession – a place where there is more time to provide care and where the logic of bureaucracy is less influential. Time is a factor which has already been highlighted as an important condition by other ethicists [53, 54]. Our contribution to the theoretical literature in this regard is that the care relationship consists of various aspects and that the four logics can be reconciled differently for each aspect of the care relationship. For each activity such as washing, feeding, leisure activities or medical services, the four logics may be balanced differently. Hence, the MLM debate benefits from dissecting the care relationship.

Our findings and previous empirical work show that for-profit nursing homes adopt a different care model from the non-profit sector [1]. For example, for-profit and non-profit nursing homes differ in their size (i.e. average number of clients) and the clients they target (i.e. socio-economic status). The different care model is a response to market incentives (also illustrated in the empirical work of Bos et al. [1]). In other words, for-profit nursing homes have adopted their distinct care model (e.g. providing small-scale nursing home sites) because the market logic dominates in the for-profit sector.

Another interesting finding is that stakeholders use different typologies to describe the for-profit nursing home sector: (i) a nursing home as a ‘home’; and (ii) nursing home as a ‘hotel’. These two different types imply different ethical considerations. Previous studies that detected the distinction between the nursing home as ‘hotel’ or ‘family home’ in their studies can help us to distil these ethical consideration from their conceptual frameworks [55, 56]. The hotel type represents a distant resident care relationship, based upon individual choice and the care recipient as empowered consumer; whereas the ‘family home’ relates to close care relationships [55, 56]. Within this typology, the nursing home as ‘hotel’ embodies more the market logic and the care home as ‘family home’ leans towards the logic of care. This variation highlights that the for-profit nursing home sector is diverse. This variation is a factor we could not explore in any depth in this study, mainly because the empirical part of this study was limited to just three for-profit nursing homes. Different financing schemes (i.e. complete reliance on private transactions versus partial private transaction) and affiliations (i.e. chain-affiliated homes versus sole proprietorship homes) could potentially affect the logics embraced by different homes. This might be an interesting subject for future research.

### The professional in the for-profit nursing home

The qualitative data illustrate that the professional in the for-profit nursing home is contrasted with the professional subject to the logic of bureaucracy. The professional in the for-profit home resists the reading of ‘care-as-discipline’.

For-profit homes are redefining the logic of professionalism. The professional in a for-profit home is, in fact, influenced by both the logic of care and the logic of the market. They may embrace of the logic of care within the context of the market for one or both of two reasons. The first reason is that the professionals adopt the logic of care in response to the demands of the care recipient. The second reason is that the conditions created by the market provide space for the professionals to build and foster those relationships with care recipients. The for-profit nursing home environment offers advantageous conditions (i.e. sufficient time per client, resources, and liberation from bureaucratic rules), allowing – at least in theory – for the adoption of the logic of care. Since this virtuous environment is created by the market, the logic of care is either way couched inside the logic of the market.

However, the extent to which the logic of care actually prevails among professionals is debatable. It can be argued that the ‘personalised care’ to which for-profit homes aspire is wrongly labelled by stakeholders. This argument holds that the care recipient is not an individual who takes part in an interdependent and equal relationship, but is instead an empowered consumer. It is difficult, from our findings, to assess whether the professional really embraces the idea of ‘the logic of care’. We can only flag this question for further research and debate.

In addition, the extent to which the professional can be autonomous when they are mainly responding to the wishes of their clients is questionable. This tension is obvious when the professional acts like the ‘activating professional’ (i.e. is trying to mentally stimulate the care recipient). The notion of the ‘activating professional’ seems to align with the idea of the professional logic of Freidson [14] but it conflicts with the ideology of consumerism – that the wishes of the care recipient dominate. This raises questions about whether the care relationship can be a mutual exchange in every circumstance, as the logic of care seem to suggest. This is an unrealistic depiction of reality and in some circumstances an undesirable relationship between the care giver and recipient. As Foucault argues, there is an inherent power divide within the relationship between physicians and patients [57].

### The residents

Our findings regarding the residents of for-profit homes yield two main conclusions. Firstly, the narratives of the residents mostly express the logic of the market: they value autonomy, customised care, the logic of choice and (negative) freedom. These values are market-related values. For-profit nursing homes seem to capitalise on these market-related desires of (prospective) care recipients. Furthermore, we found that for-profit nursing home stakeholders value the logic of choice. However, one of the ethical concerns of relying on the logic of choice – one of particular interest in this sector – is that the (prospective) care recipient needs strong social support to exercise the logic of choice effectively. In other words, you need someone who assists the (prospective) care recipient to participate in the market in order to make an informed choice. This poses serious equity concerns because this could lead to unequal access to LTC services. In addition, as Arrow [44] points out, the healthcare market does not behave as a pure market; substantial informational asymmetry between healthcare provider and healthcare recipient exists, making it a difficult for healthcare recipients to be fully informed and rational purchasers on the healthcare market.

Secondly, residents express a lack of social community within their nursing homes. Even if there is a demand for social community (and even though the for-profit nursing homes are demand-driven), it seems difficult to satisfy this demand. One of the reasons put forward by the respondents is that different social groups are placed together; the distribution mechanism of the market is based on the ability to pay and this allocation system seems to overlook the importance of social groups, religion or geography. Some entrepreneurial for-profit nursing homes in the Netherlands have recognised this limitation and tailor their nursing homes to designated social groups. For example, they have designed nursing homes specifically for specific immigrant groups (e.g. Suriname or Indonesia) or for a particular religion (e.g. Catholic or Muslim) [58]. Another limitation on the ability of for-profit homes to foster a social community is that, in a small-scale home, the chances of meeting a like-minded companion are statistically smaller than in a larger home.

### Systems thinking

With regards to the second research question, concerning which logic is dominant for each group of stakeholders, we found that when defining care, different stakeholder narratives embraced different logics. On the whole, the macro-level respondents adopted the market narrative: they commodified the care relationship to a much greater extent than respondents closer to the actual practice of care.

In discussing the desirability of market forces, the MLM debate focuses on comparisons between different sectors or different spheres of activity. When they discuss the healthcare sphere, they tend to overlook its complexity – and in particular the complexity of the care relationship. Other authors alluded to this complexity by referring to care as a polyvalent good [38], and by referring to incomplete commodification in the healthcare sector [39]. Our contribution to the MLM theoretical framework is that we emphasise instead that healthcare systems should be understood as complex systems that are shaped and formed by intermingling logics. The care relationship has various aspects and is multi-layered with the individual care relationship at the micro-level, the care organisation at the meso-level, and the healthcare system as a whole at the macro-level; at each level, different logics are prioritised. For instance, professionals can maintain their professional autonomy when the market logic is mainly manifested at the higher system level (e.g. commodification of the healthcare setting). The multi-layered system could have a filtering trickledown effect: market forces are most influential on the outer layer (the macro-level) while on the ‘lower’ levels the influence of the market becomes more diluted. A system thinking approach could enrich future research when studying the MLM. The strength of this study is that it specifies and distinguishes three different levels in the healthcare system, however, future research could refined it (e.g. distinguishing between nursing home managers and owners).

### Limitations

We used the four logics as our theoretical tools. We could, however, have opted for other frameworks. Our theoretical framework could be criticised by omitting the logic of the state. Although the state can be classified as a bureaucratic institution [59], and therefore we could claim that we did not omit the state in our framework, we would argue that the state is not necessarily equivalent to a bureaucratic organisation. In practice it often opts for this organisational logic but, theoretically, it does not have to follow the bureaucratic logic. Instead we argue that, in theory, the logic of the state corresponds to what Anderson (1990) classifies as the logic of ‘shared goods’: it is not about individual needs, wants or goods, but about providing goods on a community level. These goods are nonexclusive and, even if you cannot pay for them, they should be available. The notion of ‘shared goods’ does highlight the moral weakness of the logic of the market as distribution mechanism for LTC care since the market logic upholds the idea that people receive the good according to what they are able and willing to pay for it, which conflicts with the ideology of ‘shared goods’. A moral question which follows from this clash of logics (but which is beyond the scope of this study) is whether the market is the right tool for allocating access to social services, and, specifically related to this study, for allocating access to nursing homes with favourable conditions, which is at the moment mainly accessible for people with higher socio-economic status. (For an ethical conceptual scheme of the market as allocation method refer to Wempe and Frooman (2018) [10].) A second possible limitation of our theoretical framework is the exclusion of the logic of the family. The logic of the family allocates care based on social relationships (i.e. kinship), and there is a collective responsibility to provide the good through a reciprocal family/community [59] – often informal care [60]. Many scholars have deliberated about the ethical considerations regarding informal and institutional care [60]. However, this study focused on a particular aspect of institutional care – for-profit nursing homes – and therefore the issue of informal care and its relation to institutional care were beyond the scope of this article; hence, the logic of the family was omitted for this study.

There are several empirical limitations that might affect our findings. Firstly, we only collected qualitative data which reflects the perspective of for-profit nursing home stakeholders. Because this study was limited to the for-profit sector, we did not collect information on the perspective of the non-profit sector on their own role in the healthcare system or on the role of for-profit homes in the healthcare system. Future research should further explore this comparison. Secondly, the findings in this study could suffer from social desirability bias and choice-supportive bias. However, although we expected socially desirable answers, the respondents seem to be less affected by this factor than we expected; some respondents were surprisingly critical. Thirdly, our findings are context-dependent on the Dutch LTC sector. The for-profit sector has a distinctive role in the Netherlands and that might be an intermediating factor for our findings. The role of the for-profit nursing home sector in the Netherlands currently represents a small and parallel market to the traditional market – an opt-out option for people with more money – in contrast to the for-profit nursing homes in the United Kingdom. In the United Kingdom, for-profit nursing homes are the main LTC providers and are hugely underfunded [61]. Hence, we assume that the role of for-profit providers in LTC systems is an important factor for how for-profit nursing homes balance the different logics. The fourth and final limitation is the use of interviews (language) as our primary data for this study. Radin [62] argues that rhetoric is an important factor in how we think about morality: “Fact- and value-commitments are present in the language we use to reason and describe, and they shape our reasoning and description, and the shape (for us) of reality itself.” ([62] , p.1882). However, we found that activities and material things also matter when defining ‘good’ care. In order to take into account how materialities contribute in shaping realities, future studies could follow the material semiotic approach, as has been proposed by Pols [63] and Driessen [64]. Both scholars refer to this as the radicalisation of relationality, meaning “that things, activities and words are added to the study of relations between people” ([63] , p.176).

## Conclusion

The for-profit nursing home sector embrace the logic of the market but the for-profit nursing home sector reconcile the market logic with the logic of care and the logic of professionalism. The market logic is present in the for-profit nursing home sector because these nursing homes revolve around the demands of the residents. On the other hand, the for-profit sector does create an environment for professionals to provide person-oriented care.

We identify four lessons learned from this empirical ethical research project for the MLM debate. Firstly, the provision of care should not be treated as one unit in the MLM debate, as it has often been. Each and every aspect of care should be considered. For each aspect, the market logic is reconciled with competing logics in different ways: whereas the nursing home setting is commodified, the care relationship is much less so. Secondly, a multilevel approach is necessary for assessing the influence of the market in healthcare systems. The market logic is mostly expressed by respondents at the macro-level, whereas people closer to the care relationship seem to prioritise and embrace other logics. Thirdly, respondents describe the for-profit nursing home professionals as the antithesis of the bureaucratic professional, and, in practice, the for-profit sector seeks to create a new professional logic that resembles the logic of care. It seeks to do this by creating an environment with favourable conditions (i.e. enough time to provide care and resources) which should enable caregivers to maintain their professional integrity. Nevertheless, the professional logic is also ultimately driven by the market logic: they must first and foremost respond to residents’ wishes. Hence, it is difficult to characterise clearly the professionals as embracing the logic of care rather than the logic of the market. Lastly, the residents express several market-related values, such as autonomy, customised care and (negative) freedom.

## Supplementary Information

**Additional file 1.**

**Additional file 2.**

## Data Availability

The qualitative data is available from the authors upon reasonable request and restriction apply (e.g. acquiring permission of the participants).

## Abbreviations

LTC: Long-term care
MLM: Moral Limits of Markets
