# Supplementary material for: How the logics of the market, bureaucracy, professionalism and care are reconciled in practice: an empirical ethics approach
Source: BMC Health Serv Res. 2020 Nov 10;20:1024. doi: 10.1186/s12913-020-05870-7 (PMC7654039; doi:10.1186/s12913-020-05870-7)
Supplement: Supplementary file 1 — Additional file 1. [file 12913_2020_5870_MOESM1_ESM.docx]

**Additional File A**

In 2015, the Netherlands introduced a LTC system reform, driven by the concern about the financial sustainability of the sector. An important goal of this this reform was to support and stimulate people to stay at home longer and organise care in the home-setting (Maarse and Jeurissen 2016). Among other things, the LTC reform boosted also the creation of a private market for individuals to choose and organise their own care. This was a result of retrenchment strategies and possibilities to combine public benefits with the private money of well-to-do people that seek care.

There exists no public ownership in the Dutch LTC sector, and since for-profit ownership is prohibited for intramural care services that exceeds 6 beds (The Ministry of Health Welfare and Sport 2005), the private non-profit sector has been overwhelmingly dominant. Nevertheless, the for-profit sector recently found a way to position itself and currently 12% of nursing homes are for-profit, although they mostly operate with comparatively smaller scales (Bos et al. 2020). As a result, the share of the total for-profit nursing home client population is relatively small: approximately 4% lives in for-profit nursing homes (Bos et al. 2020).

For-profit nursing homes are able to ‘avoid’ the ban by separating the fees for housing, care and extra amenities. They provide clustered living arrangements that can be financed in two ways. First, for-profit nursing homes can be financed through in-kind extramural packages called the total home-care package. These are publicly funded benefits, although income related co-payments do apply. The care recipients then have to privately pay rents to the for-profit nursing home for their residencies and might consume other services as well. Second, the for-profit nursing home can be financed through publicly funded personal budgets. When the nursing home is financed through personal budgets, both the care and living arrangements become a private transaction between the nursing home and the care recipient. Both the uptake for total home-care package and personal budgets increased, 66% and 51% respectively from 2015 to 2018, while the uptake for in-kind intramural packages fell recently with -1% from 2015 to 2018 (Statistics Netherlands (CBS) 2019).

Residents of higher socio-economic status have to pay high obligatory co-payments for living in in-kind intramural (non-profit) LTC homes. These depend on their income and also means testing does apply. This has helped to create a market for for-profit nursing homes to attract clients from a higher socio-economic background who, either way, have to pay a significant amount in the traditional non-profit sector.

The for-profit nursing homes are often small-scale houses with a maximum of 25 clients. Traditional non-profit nursing homes are generally much larger (Plaisier and den Draak 2019).

**References**

Bos, Aline, Florien M. Kruse, and Patrick P.T. Jeurissen. 2020. For-profit nursing homes in the Netherlands: what factors explain their rise? *International Journal of Health Services (forthcoming)*.

Maarse, Hans, and Patrick P.T. Jeurissen. 2016. The policy and politics of the 2015 long-term care reform in the Netherlands. *Health Policy* 120 (3):241-245. <https://doi.org/10.1016/j.healthpol.2016.01.014>.

Plaisier, Inger, and Maaike den Draak. 2019. Wonen met Zorg. Verkenning van particuliere woonzorg voor ouderen. The Hague: Sociaal en Cultureel Planbureau (SCP) [The Netherlands Institute for Social Research].

Statistics Netherlands (CBS). 2019. [Dataset] Personen met indicatie naar gebruik Wlz-zorg; indicatie, leveringsvorm, zzp.

The Ministry of Health Welfare and Sport. 2005. Uitvoeringsbesluit WTZi.
