# Supplementary material for: How the logics of the market, bureaucracy, professionalism and care are reconciled in practice: an empirical ethics approach
Source: BMC Health Serv Res. 2020 Nov 10;20:1024. doi: 10.1186/s12913-020-05870-7 (PMC7654039; doi:10.1186/s12913-020-05870-7)
Supplement: Supplementary file 2 — Additional file 2. [file 12913_2020_5870_MOESM2_ESM.docx]

**Additional File B**

Resident or family member of the resident

| Wat vindt u goede zorg?  [Wat is belangrijk voor u bij de dagelijkse zorg?] | How would you define good care?  [Supporting question: what is according to you important in providing care] |
| --- | --- |
| Krijgt u de zorg die u belangrijk vindt ? Waarom wel of niet?  [Terug refereren naar de vorige vraag en hun antwoord + vragen om te illustreren met een voorbeeld] | Do you receive the care that is important to you? Why do or don’t you receive this care?  [Supporting question: Refer back to their answers to the previous question and ask for concrete examples] |
| Wat voor activiteiten [naast slapen, eten, wassen] onderneemt u nog meer op een dag?  [En hoe is dit gelinkt aan het concept ‘goede zorg’] | Besides sleeping, eating and washing, what kind of activities do you do during the course of the day?  [Supporting question: How does this relate to their definition of good quality of care?] |
| Waarom heeft u voor dit huis gekozen ? Wat is inderdaad zo en wat is er anders dan u verwacht had? | Why did you choose this nursing home? How does it fit your expectations? |
| Kunt u het contact tussen u en de verzorgende, medebewoners, medewerkers en bestuurder(s) omschrijven? | How would you describe the contact between you and the care professionals, the other residents, the other employees working here and the nursing home manager? |
| Wat voor soort mensen wonen er in dit huis? Wat voor soort mensen werken er in dit huis?  [Hoe zou u ze typeren?] | How would you describe the kind of people living in this nursing home? And could you describe what kind of people work in this home?  [Supporting questions: How would you typify the people living in this nursing home? ] |
| Waar draait het volgens u om in dit huis? | What is according to you the objective of this nursing home? |
| Wat is de zorgvisie van dit huis?  [Wat is volgens u het doel dat dit huis nastreeft? Is er 1 doel of zijn er meerdere doelen?] | How would you describe the vision of this home?  [Support questions: What is, according to you, the goal that this house pursues? Are there one or multiple goals? ] |
| Zijn er nog andere dingen waar u het over wilt hebben? | Are there other aspects that you would like to discuss with us in the light of this study? |

Healthcare professional

| Wat vindt u goede zorg?  [Wat vindt u belangrijk in het verlenen van zorg?] | How would you define good care?  [Supporting question: what is, according to you, important in providing care] |
| --- | --- |
| Lukt het om deze zorg te leveren? Waarom wel of niet?  [Terug refereren naar de vorige vraag en hun antwoord + vragen om te illustreren met een voorbeeld] | Are you able to provide this quality of care? Why are or aren’t you able to do so?  [Supporting question: Refer back to their answers to the previous question and ask for concrete examples] |
| Wat voor activiteiten [naast slapen, eten, wassen] onderneemt u nog meer op een dag?  [En hoe is dit gelinkt aan het concept ‘goede zorg’] | Besides the daily activities such as sleeping, eating and washing, what else do you do during the course of the day?  [Supporting question: how does this relate to their definition of good quality of care?] |
| Waarom heeft u voor dit beroep gekozen? | Why did you choose this profession? |
| Waarvoor voelt u zich verantwoordelijk en waarvoor niet? | In how far do you feel responsible for care in this home? Where does your feeling of responsibility ends? |
| Kunt u het contact tussen u en de bewoners omschrijven?  [naar naasten vragen, doorvragen zodat echt verhouding duidelijk wordt] | How would you describe the contact between you and the residents?  [Supporting question, in case it wasn’t mentioned: How is your relationship with the residents’ family or their social support?] |
| Wat voor soort mensen wonen er in dit huis? Wat voor soort mensen werken er in dit huis?  [Hoe zou u ze typeren? En hoe komen de bewoners hier terecht?] | How would you describe the kind of people living in this nursing home? And could you describe what kind of people work in this nursing home?  [Supporting question: How would you typify the people living in this nursing home? How do most residents gain access to this nursing homes?] |
| Waarom werkt u specifiek voor deze zorgorganisatie? | Why do you specifically work for this healthcare organisation? |
| Wat is de zorgvisie van dit huis?  [Wat is volgens u het doel dat dit huis nastreeft? Is er 1 doel of zijn er meerdere doelen?] | How would you describe the vision of this home?  [Support questions: What is, according to you, the goal that this nursing home pursues? Are there one or multiple goals? ] |
| Zijn er nog andere dingen waar u het over wilt hebben? | Are there other aspects that you would like to discuss with us in the light of this study? |

Nursing home manager

| Wat vindt u goede zorg?  [Wat vindt u belangrijk in het verlenen van zorg?] | How would you define good care?  [Supporting question: what is, according to you, important in providing care] |
| --- | --- |
| Lukt het om deze zorg te leveren? Waarom wel of niet?  [Terug refereren naar de vorige vraag en hun antwoord + vragen om te illustreren met een voorbeeld] | Are you able to provide this quality of care? Why are or aren’t you able to do so?  [Supporting question: Refer back to their answers to the previous question and ask for concrete examples] |
| Wat voor activiteiten [naast slapen, eten, wassen] onderneemt u nog meer op een dag?  [En hoe is dit gelinkt aan het concept ‘goede zorg’] | Besides the daily activities such as sleeping, eating and washing, what else do you do during the course of the day?  [Supporting question: how does this relate to their definition of good quality of care?] |
| Wat is uw motivatie om leiding te geven aan dit huis? Waarom bent u dit huis gestart of waarom bent u hier gaan werken? | What motivates you to manage this nursing home? Why did you establish this nursing home or why did you decide to work here? |
| Kunt u het contact tussen u en de bewoners omschrijven?  [Naar naasten vragen, doorvragen zodat echt verhouding duidelijk wordt] | How would you describe the contact between you and the residents?  [Supporting question, in case it wasn’t mentioned: How is your relationship with the residents’ family or their social support?] |
| Wat voor soort mensen wonen er in dit huis? Wat voor soort mensen werken er in dit huis?  [Hoe zou u ze typeren? En hoe komen de bewoners hier terecht?] | How would you describe the kind of people living in this nursing home? And could you describe what kind of people work in this nursing home?  [Supporting question: How would you typify the people living in this nursing home? How do most residents gain access to this nursing homes?] |
| Wat is de zorgvisie van dit huis?  [Wat is volgens u het doel dat dit huis nastreeft? Is er 1 doel of zijn er meerdere doelen?] | How would you describe the vision of this home?  [Support questions: What is according to you the goal that this house pursues? Are there one or multiple goals?] |
| Wat vindt u belangrijk als u denkt aan de zorg voor ouderen in Nederland? En waarom? | What is, according to you, important for elderly care in the Netherlands? And why? |
| Zijn er nog andere dingen waar u het over wilt hebben? | Are there other aspects that you would like to discuss with us in the light of this study? |

Expert

| Hoe duidt u de groei van particuliere verpleeghuizen over de afgelopen jaren? | How do you interpret the growth of for-profit nursing homes in the recent years? |
| --- | --- |
| Welke kansen ziet u voor deze sector? | What are, according to you, the opportunities for this sector? |
| Welke belemmeringen ziet u voor deze sector? | What are, according to you, the challenges for this sector? |
| Waarin verschillen particuliere verpleeghuizen in uw optiek van reguliere verpleeghuizen? | In your view, how do for-profit nursing homes differ from regular nursing homes |
| Denkt u dat particuliere verpleeghuizen meer onderdeel van de verpleeghuiszorg (*mainstream*) gaan worden in de toekomst? Zo ja waarom wel en waarom niet? | Do you think that for-profit nursing homes will become mainstream in the future? If so, why so and why not? |
